# Decreasing Cell Population of Individual *Candida* Species Does Not Impair the Virulence of *Candida albicans* and *Candida glabrata* Mixed Biofilms

**DOI:** 10.3389/fmicb.2019.01600

**Published:** 2019-07-11

**Authors:** Qianqian Li, Juanjuan Liu, Jing Shao, Wenyue Da, Gaoxiang Shi, Tianming Wang, Daqiang Wu, Changzhong Wang

**Affiliations:** ^1^Laboratory of Pathogenic Biology and Immunology, College of Integrated Chinese and Western Medicine (College of Life Science), Anhui University of Chinese Medicine, Hefei, China; ^2^Institute of Integrated Traditional Chinese and Western Medicine, Anhui University of Chinese Medicine, Hefei, China; ^3^Key Laboratory of Xin’an Medicine, Ministry of Education, Anhui University of Chinese Medicine, Hefei, China

**Keywords:** *Candida albicans*, *Candida glabrata*, mixed biofilm, dual-species biofilm, efflux pump, β-glucan, virulence

## Abstract

*Candida albicans* and *Candida glabrata* are two commonly seen opportunistic fungi in clinical settings and usually co-isolated from the population inflicted with denture stomatitis and oropharyngeal candidiasis. Although *C. albicans* and *C. glabrata* mixed biofilm is deemed to possess enhanced virulence compared with their individual counterparts (especially *C. albicans* single biofilm), the relevant descriptions and experimental evidence on the relationship of *Candida* virulence with their individual cell number in mixed biofilms are contradictory and insufficient. In this study, two standard *C. glabrata* isolate and eight *C. albicans* ones were used to test the cell quantities in their 24- and 48-h single and mixed biofilms. A series of virulence factors including antifungal resistance to caspofungin, secreted aspartic proteinase (SAP) and phospholipase (PL) levels, efflux pump function and β-glucan exposure were evaluated. Through this study, the declines of individual cell counting were observed in the 24- and 48-h *Candida* mixed biofilms compared with their single counterparts. However, the antifungal resistance to caspofungin, the SAP and phospholipase levels, the rhodamine 6G efflux and the efflux-related gene expressions were increased significantly or kept unchanged accompanying with reduced β-glucan exposure in the mixed biofilms by comparison with the single counterparts. These results reveal that there is a competitive interaction between *C. albicans* and *C. glabrata* strains in their co-culture without at the expense of the mixed biofilm virulence. This study presents a deep insight into the interaction between *C. albicans* and *C. glabrata* and provides new clues to combat against fungal infections caused by *Candida* mixed biofilms.

## Introduction

Among the pathogenic fungi, *Candida* species are undoubtedly the major etiological agents that is capable of inducing a variety of uncomfortable or even deadly complications (Arendrup and Patterson, 2017). They are prevalent in nearly 70% of healthy population and distributed in many niches such as oral cavity, gastrointestinal tract, urogenital tract, and skin (Gow et al., 2012). Mounting evidence have demonstrated that *Candida* species can interact with each other with two main presentations including commensalism and antagonism (Thein et al., 2009). One of the critical features of *Candida*–*Candida* interaction is the ability to form biofilm which is a protective consortia of encased microorganisms by extracellular matrix from external disturbs. It is acknowledged that biofilm communities can evolve more resistance to conventional drugs (azoles, polyenes, echinocandins) than their planktonic counterparts (Thein et al., 2009; Peleg et al., 2010).

In clinical context, *Candida albicans* and *Candida glabrata* are believed to be the first and second/third most isolated fungi from the severe immunosuppressed or immunocompromised individuals, such as the patients with long-range antibiotics/antifungals therapy, HIV/AIDS and tumor patients (Silva et al., 2012; Sardi et al., 2013; Rodrigues et al., 2014). Their morbidities and mortalities might be diverse in different regions and races, but they are reported to be the leading manipulators of candidiasis and candidemia (Pfaller and Diekema, 2007; Wisplinghoff et al., 2014). *C. albicans* and *C. glabrata* are often coisolated from populations associated with denture stomatitis (Coco et al., 2008) and oropharyngeal candidiasis (Tati et al., 2016). Some studies demonstrated that there was a synergism between *C. albicans* and *C. glabrata*, and what is more, the pre-colonization of *C. albicans* facilitated following proliferation of *C. glabrata* in their mixed biofilms (Pathak et al., 2012; Silva et al., 2013). This mutually beneficial collaboration might be largely due to the “complementary” morphologies as yeast-form *C. glabrata* can attach to *C. albicans* hyphae which promotes the fungal cells to penetrate mucosal surfaces of host tissue (Tati et al., 2016).

The interspecific relative abundance is monitored by host immune system and might keep constant during internal proliferation. Once the balance is broken, dysbiosis occurs leading to microbial invasions and diseases. Although most studies showed that the co-culture of *C. glabrata* and *C. albicans* can improve their invasions into host tissue compared with their single cultures (Coco et al., 2008; Pathak et al., 2012; Alves et al., 2014; Tati et al., 2016), the reports on the competitive advantages of both *Candida* species during their co-culture and their mixed biofilm virulence compared with the single counterpart are contradictory (Silva et al., 2013; Rossoni et al., 2015; Hosida et al., 2018; Olson et al., 2018). Thus, it is consequential to illuminate the relationship between the individual *Candida* abundance and the virulence in *C. albicans* and *C. glabrata* mixed biofilms.

In this study, we employed eight *C. albicans* and two *C. glabrata* strains to form 24- and 48-h *C. albicans* and *C. glabrata* single and mixed biofilms. Each species cell number was quantified and a group of virulence factors including antifungal resistance to caspofungin, secreted protease levels, efflux pump functions and β-glucan exposure were monitored. Based on these results, we explored the role of the *Candida* proportion in mediating the virulence of single and mixed biofilms.

## Materials and Methods

### Fungal Strains and Cultivations

*Candida albicans* SC5314 was kindly provided by Prof. Yuanying Jiang from College of Pharmacy, the Second Military Medical University (Shanghai, China). Seven clinical *C. albicans* isolates including Z3044, Z2003, Z1402, Z1407, Z826, Z103, and Z215 were donated by Huaiwei Lu, Clinical Laboratory, Anhui Provincial Hospital (Hefei, China). *C. glabrata* ATCC15126 was acquired from National Institutes for Food and Drug Control (Beijing, China). All of the *Candida* strains were confirmed by CHROmagar medium (Shanghai, China) prior to experiments. Based on The strains were revived in liquid Sabouraud medium (Hope Biotech., Co., Qingdao, China) at 37°C for 12–16 h till the exponential phase and collected at 3000 g (Leiboer Medical Devices, Beijing, China) followed by twice washings with sterile phosphate-buffered saline (PBS, 0.01M, pH 7.2, Leagene, Beijing, China). The fungal cells were then resuspended in RPMI-1640 medium (pH 7.0, Invitrogen, Carlsbad, CA, United States) and adjusted to a proper cell density using a hemocytometer prior to the experiments (Wang et al., 2018).

### Biofilm Formation

*Candida* static biofilms were formed on PVC catheters according to our previous work with mild modifications (Shao et al., 2015). In brief, the single and mixed *Candida* biofilms were formed on pre-sterilized 3 cm × 0.5 cm (long × width) grooved polyvinyl chloride (PVC) catheters (Shuguang Jianshi, Luohe, China). A quantity of *Candida* cells (= 5 × 10^4^ CFU/mL for both *C. albicans* and/or *C. glabrata*) was resuspended in RPMI-1640 (pH 7.0) at 37°C for 4 h. Following the initial attachment, the catheters were washed twice with sterile H_2_O and immersed in an incubator containing 40 mL RPMI-1640 (pH 7.0) for another 20 and 44 h of incubation at 37°C. The ratio of *C. albicans* and *C. glabrata* for mixed biofilm formation was 1:1 (Shao et al., 2015).

### Susceptibility Test

The procedures of broth microdilution method and XTT analysis were referenced previously with a few modifications (Ramage et al., 2001). The initial concentration was 5 × 10^4^ CFU/mL for *Candida* single and mixed biofilm formations. The stock solution of caspofungin (Yuanye, Shanghai, China) was prepared by direct dissolving the drug into RPMI-1640 (pH 7.0) to the concentration of 100 μg/mL. To test sessile minimum inhibitory concentration (SMIC), the caspofungin storage underwent a series of twofold dilution. If the end-point appeared between two dilutions, a further dilution will performed till the change between two concentrations was 0.05 μg/mL. The SMIC_80_ was defined as the drug concentration that inhibited 80% of fungal biofilms by comparing the metabolic activity with that of drug-free control at 492 nm using XTT (Sangon, Shanghai, China) method.

### Cell Counting

The preformed 24- and 48-h single and dual *Candida* biofilms were disaggregated from the PVC catheter for successively 1 min per each time of vortexing (Quick Mixer SK-1, Guowang Experimental Instrument Factory, Jintan, China) for three times and 20 min of 50 kHz gentle sonication (DSA50-GL1, Desen Ultrasonic Equipment, Fuzhou, China) at an amplitude of 0.45–0.55 w/cm^2^ at room temperature according to a previous work with a few modifications (Zarnowski et al., 2014). The harvested fungal cells were resuspended in RPMI-1640 medium (pH 7.0) and serially 10-fold diluted. The dilutions were spread onto CHROmagar plates at 37°C for 24 h and the cell quantity was recorded as colony forming unit per milliliter per catheter accordingly.

### Spot Assay

The procedures were based on the steps of a previous report (Mukhopadhyay et al., 2002). Briefly, the 24- and 48-h preformed single and dual *Candida* biofilms were disaggregated as described above. The fungal cells were resuspended and diluted by RPMI-1640 medium (pH 7.0) into the final concentrations of 1 × 10^4^ and 1 × 10^5^ CFU/mL. Five microliters of each prepared single and dual *Candida* culture was spotted onto Sabouraud plates in the presence of 0.2 and 0.3 μg/mL caspofungin and incubated for 24 h at 37°C.

### Biofilm Quantification

The total biomass of single and mixed *Candida* biofilms was quantified by crystal violet (CV, Macklin, Shanghai, China) and the experimental procedures were performed with moderate modification as previously described (Hosida et al., 2018). Briefly, 100 μL strain-contained culture and 100 μL caspofungin-included medium were added into a 96-well plate at 37°C for 24 and 48 h. Following the formation of *Candida* biofilms, the supernatant was removed and 200 μL of 99% methanol (Suyi, Shanghai, China) for fixation at room temperature for 15 min. The methanol was then discarded and the fixed *Candida* biofilms were allowed to dry and incubated with 200 μL of 1% pre-filtered CV for 5 min. After the removal of CV, the wells were gently rinsed twice with deionized water, and 200 μL of 33% acetic acid (Xilong, China) was then pipetted into each well for CV release. The absorbance values were surveyed at 562 nm. The blank control was drug-free wells containing RPMI-1640 (pH 7.0) only.

### Hydrolytic Enzyme Detection

The 24- and 48-h preformed single and dual *Candida* biofilms were disaggregated as described above. The supernatant was pooled for the measurement of secreted aspartic proteinase (SAP, cat. MM-230001) and phospholipase B1 (PLB1, cat. MM-229001) with commercial ELISA kits (Meimian, Shanghai, China). The experimental procedures of ELISA were performed according to the instructions.

### Rhodamine 6G (R6G) Efflux Assay

The experiment was performed according to the procedures described previously with a few modifications (Wang et al., 2018). Briefly, each single and dual *Candida* culture (= 5 × 10^4^ CFU/mL) was employed for the 24- and 48-h biofilm formations. The supernatant was discarded with 3000 *g* centrifugation for 3 min and the remnants were mixed with 2 mL sterile PBS (0.01M, pH 7.2) including 2 mM glucose for 20 min at 37°C. After 3000 *g* centrifugation for 3 min, a quantity of rhodamine-6G (100 μL, 10 μM, Macklin, Shanghai, China) was then added for 2 h in the darkness. The supernatant was then discarded and the pellets were washed three times by sterile PBS (0.01M, pH 7.2). The fluorescent image was observed with an inverted fluorescence microscope IX71 (Olympus, Tokyo, Japan) and the fluorescent intensity was measured with BD Accuri^TM^ C6 flow cytometer (Shanghai, China) at 488 nm excitation wavelength and 525 nm emission wavelength.

### RNA Extraction and qRT-PCR Analysis

The preformed 24- and 48-h single and dual *Candida* biofilm cells were collected by 3000 g. Total RNA samples were extracted according to the instructions of MagExtractor-RNA kit (ToyoBo, Tokyo, Japan) with OD260/OD280 = 1.8–2.0. Six microliters of the extracted RNA was incubated with 2 μL 4 × DNA Master I (containing gDNA Remover) and 2 μL 5RT-Master Mix II, and reverse-transcribed into cDNA as recommended by ReverTra Ace qPCR RT Master Mix with gDNA Remover kit (ToyoBo, Tokyo, Japan) with procedures as follows: 65°C for 5 min and 4°C for 1 min for initial RNA denaturation, followed by 37°C for 15 min, 50°C for 5 min, and 4°C for 1 min. The prepared cDNA was diluted 10-fold (approximate 1 ng/μL) prior to RT-PCR. Primers for *C. albicans* and *C. glabrata* could be referenced to a previous study (Wang et al., 2018). Twenty-five microliter of real time PCR mixture was freshly prepared containing 12.5 μL of 2 × SYBR Green Realtime PCR, 1 μL of PCR Forward Primer, 1 μL of PCR Reverse Primer, 0.5 μL of cDNA, and 10 μL of ddH_2_O. The PCR process were performed on ABI7000 fluorescent quantitative PCR system (Applied Biosystem) with following cycles: 95°C for 60 s for pre-denaturation alone with 95°C for 15 s, 55°C for 15 s, 72°C for 45 s for a total of 40 cycles. All data were normalized to housekeeping gene ACT1 as the internal reference gene (Wang et al., 2018). The relative target-gene expression was calculated as a fold change of 2^–ΔΔ*Ct*^ value, in which ΔC_t_ = C_t_^*targetgene*^ – C_t_^internal^ ^referencegenes^ as previously described (Livak and Schmittgen, 2001). The *CDR1* mRNA of the 24-h *C. albicans* SC5314 single biofilm was set as the control.

### β-Glucan Exposure Test

The unmasking procedures were conducted as instructed with a few adjustments (Sherrington et al., 2017). To stain for surface exposed β-1,3-glucan, the preformed 24- and 48-h single and dual *Candida* biofilm cells were blocked with 2% BSA Albumin Fraction V (Biofroxx, Shanghai, China) in PBS (0.01M, pH 7.2) for 1 h and then incubated for 4 h with a monoclonal anti-β-1,3-glucan antibody (Bioscience Supplies, Australia) diluted 1:300 in PBS (0.01M, pH 7.2) at 4°C with gentle shaking. Followed by primary antibody treatment, cells were washed three times with PBS (0.01M, pH 7.2) and incubated with 1:100 diluted goat anti-mouse IgG conjugated to Cy3 (Abbkine, Shanghai, China) for 1 h at 4°C. Cells were pooled by 3000 *g* centrifugation for 5 min after washing twice with sterile PBS (0.01M, pH 7.2). The fluorescent intensity was measured with BD Accuri^TM^ C6 flow cytometer at 488 nm excitation wavelength and 570 nm emission wavelength.

### Statistical Analysis

All procedures were performed in triplicate in three times on three occasions. The results were reported as mean ± standard deviation, calculated by SPSS 17.0, and processed by one-way ANOVA with least significant difference (LSD) method. The comparison among groups adopted Student’s *t*-test. The statistical significance was defined as *p* < 0.05.

## Results

### Relative Abundance of Single/Mixed *C. albicans* and *C. glabrata* Biofilms

**FIGURE 1:**
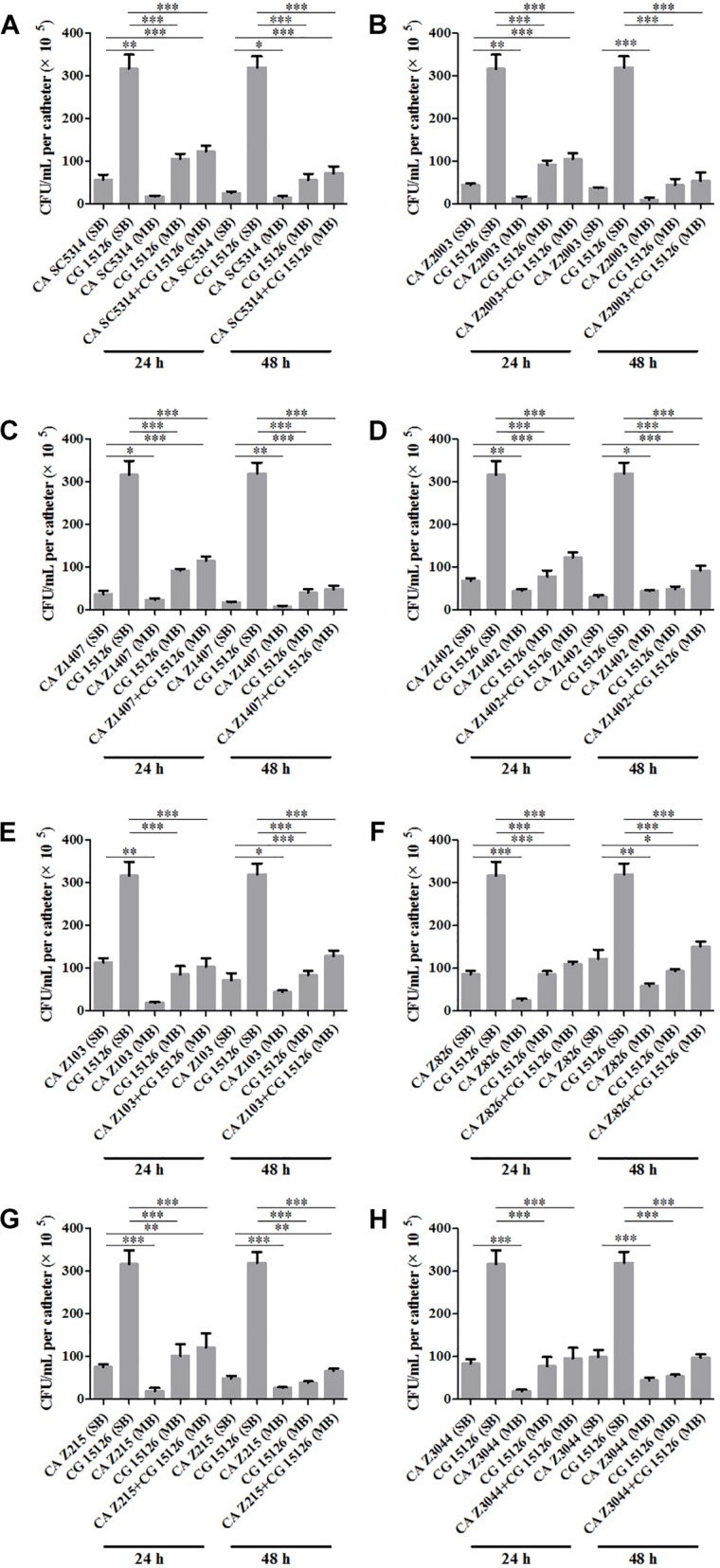
Cell counting in the 24- and 48-h **(A)** *Candida albicans* SC5314 (CA SC5314) and *Candida glabrata* ATCC15126 (CG), **(B)** *C. albicans* Z2003 (CA Z2003)and *C. glabrata* ATCC15126 (CG), **(C)** *C. albicans* Z1407 (CA Z1407)and *C. glabrata* ATCC15126 (CG), **(D)** *C. albicans* Z1402 (CA Z1402)and *C. glabrata* ATCC15126 (CG), **(E)** *C. albicans* Z103 (CA Z103) and *C. glabrata* ATCC15126 (CG), **(F)** *C. albicans* Z826 (CA Z826)and *C. glabrata* ATCC15126 (CG), **(G)** *C. albicans* Z215 (CA Z215)and *C. glabrata* ATCC15126 (CG), **(H)** *C. albicans* Z3044 (CA Z3044) and *C. glabrata* ATCC15126 (CG) single and mixed biofilms. ^*^p < 0.05; ^∗∗^*p* < 0.01; ^∗∗∗^*p* < 0.001. SB, single biofilm; MB, mixed biofilm.

**FIGURE 2:**
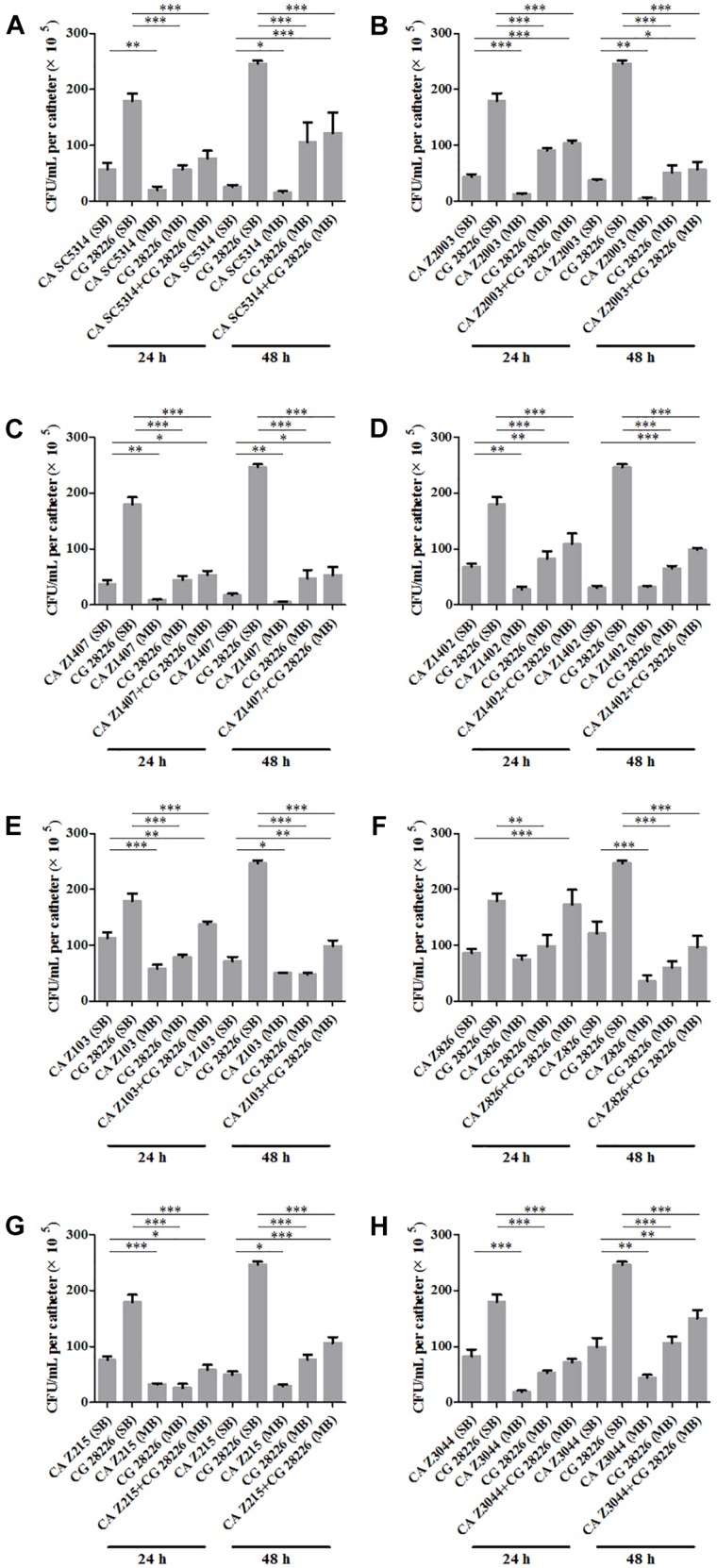
Cell counting in the 24- and 48-h **(A)** *C. albicans* SC5314 (CA SC5314) and *C. glabrata* ATCC28226 (CG), **(B)** *C. albicans* Z2003 (CA Z2003) and *C. glabrata* ATCC28226 (CG), **(C)** *C. albicans* Z1407 (CA Z1407) and *C. glabrata* ATCC28226 (CG), **(D)** *C. albicans* Z1402 (CA Z1402) and *C. glabrata* ATCC28226 (CG), **(E)** *C. albicans* Z103 (CA Z103) and *C. glabrata* ATCC28226 (CG), **(F)** *C. albicans* Z826 (CA Z826) and *C. glabrata* ATCC28226 (CG), **(G)** *C. albicans* Z215 (CA Z215) and *C. glabrata* ATCC28226 (CG), **(H)** *C. albicans* Z3044 (CA Z3044) and *C. glabrata* ATCC28226 (CG) single and mixed biofilms. ^*^p < 0.05; ^∗∗^*p* < 0.01; ^∗∗∗^*p* < 0.001. SB, single biofilm; MB, mixed biofilm.

Eight *C. albicans* and two *C. glabrata* strains were used to explore the cell populations in the single and the 24- and 48-h mixed biofilms. When combining *C. glabrata* ATCC15126 with the eight *C. albicans* strains, whether at 24 or 48 h, the *C. albicans* cell populations in the single biofilms were all significantly higher than those in its mixed counterpart (Figure 1), and showed remarkable decrease in most cases compared with the entire cell number in the corresponding mixed biofilms except *C. albicans* Z2003 (48 h, Figure 1B), Z103 (24 h, Figure 1E), and Z3044 (24 and 48 h, Figure 1H). Meanwhile, the growth of *C. glabrata* ATCC15126 were all dramatically potent compared with its mixed counterpart (*p* < 0.001, Figure 1). In combination of *C. glabrata* ATCC28226 with the same eight *C. albicans* strains, the similar observations could also be obtained with several changes. These changes included that *C. albicans* SC5314 and Z3044 cell populations did not show advantages over the total cell number in the corresponding 24-h mixed biofilms (Figures 2A,H), and the cell quantities of the 24-h *C. albicans* Z826 single biofilm and *C. glabrata* ATCC28226 single biofilm exhibited no significant difference compared with the individual *C. albicans* cell number and the gross cell counting of the corresponding mixed counterpart (Figure 2F). Meanwhile, the development of *C. albicans* Z826 single biofilm also exhibited no superiority to its corresponding 48-h mixed counterpart (Figure 2F). In view of relative abundance of each *Candida* strains, the majority of cell proportion trends (12/16 for *C. glabrata* ATCC15126 and its combinations, 11/16 for *C. glabrata* ATCC28226 and its combinations) underwent elevations. The trends showing decline (4/16 in both *C. glabrata* strains and their combinations) mainly appeared in the 24-h biofilms (Tables 1, 2).

**TABLE 1:** Relative abundance of individual *C. albicans* species and *C. glabrata* ATCC15126 in their 24- and 48-h single biofilms (SB) and mixed biofilms (MB).

	**Cell proportion (Mean CFU of *C. albicans* : Mean CFU of *C. glabrata*)**					
**Strains**	**SB (24 h)**	**MB (24 h)**	**Trend (24 h)^#^**	**SB (48 h)**	**MB (48 h)**	**Trend (48 h)^#^**
*C. albicans* SC5314/*C. glabrata* ATCC15126	1:5.52	1:6.10	Decrease	1:12.62	1:3.63	Increase
*C. albicans* Z2003/*C. glabrata* ATCC15126	1:7.25	1:6.35	Increase	1:8.64	1:4.37	Increase
*C. albicans* Z1407/*C. glabrata* ATCC15126	1:8.56	1:3.99	Increase	1:18.8	1:5.26	Increase
*C. albicans* Z1402/*C. glabrata* ATCC15126	1:4.61	1:1.72	Increase	1:10.54	1:1.08	Increase
*C. albicans* Z103/*C. glabrata* ATCC15126	1:2.80	1:4.55	Decrease	1:4.48	1:1.90	Increase
*C. albicans* Z826/*C. glabrata* ATCC15126	1:3.7	1:3.46	Increase	1:2.63	1:1.61	Increase
*C. albicans* Z215/*C. glabrata* ATCC15126	1:4.18	1:5.37	Decrease	1:6.48	1:1.43	Increase
*C. albicans* Z3044/*C. glabrata* ATCC15126	1:3.83	1:4.32	Decrease	1:3.23	1:1.21	Increase

*^#^The trend represents decrease (gray grid)/increase (white grid) of the fungal cell proportions (*C. albicans* : *C. glabrata*) via comparing the SB with the MB at 24 and 48 h respectively.*

**TABLE 2:** Relative abundance of individual *C. albicans* species and *C. glabrata* ATCC28226 in their 24- and 48-h single biofilms (SB) and mixed biofilms (MB).

	**Cell proportion (*C. albicans* : *C. glabrata*)**					
**Strains**	**SB (24 h)**	**MB (24 h)**	**Trend (24 h)^#^**	**SB (48 h)**	**MB (48 h)**	**Trend (48 h)^#^**
*C. albicans* SC5314/*C. glabrata* ATCC28226	1:3.13	1:2.77	Increase	1:9.71	1:6.76	Increase
*C. albicans* Z2003/*C. glabrata* ATCC28226	1:4.11	1:7.61	Decrease	1:6.65	1:9.63	Decrease
*C. albicans* Z1407/*C. glabrata* ATCC28226	1:4.85	1:5.15	Decrease	1:14.47	1:8.88	Increase
*C. albicans* Z1402/*C. glabrata* ATCC28226	1:2.61	1:3.04	Decrease	1:8.11	1:2.03	Increase
*C. albicans* Z103/*C. glabrata* ATCC28226	1:1.59	1:1.35	Increase	1:3.44	1:0.95	Increase
*C. albicans* Z826/*C. glabrata* ATCC28226	1:2.09	1:1.33	Increase	1:2.03	1:1.67	Increase
*C. albicans* Z215/*C. glabrata* ATCC28226	1:2.37	1:0.78	Increase	1:4.99	1:2.61	Increase
*C. albicans* Z3044/*C. glabrata* ATCC28226	1:2.17	1:2.87	Decrease	1:2.48	1:2.41	Increase

*^#^The trend represents decrease (gray grid)/increase (white grid) of the fungal cell proportions (*C. albicans* : *C. glabrata*) via comparing the SB with the MB at 24 and 48 h respectively.*

### Antifungal Effects of Caspofungin on Single/Mixed *C. albicans* and *C. glabrata* Biofilms

**FIGURE 3:**
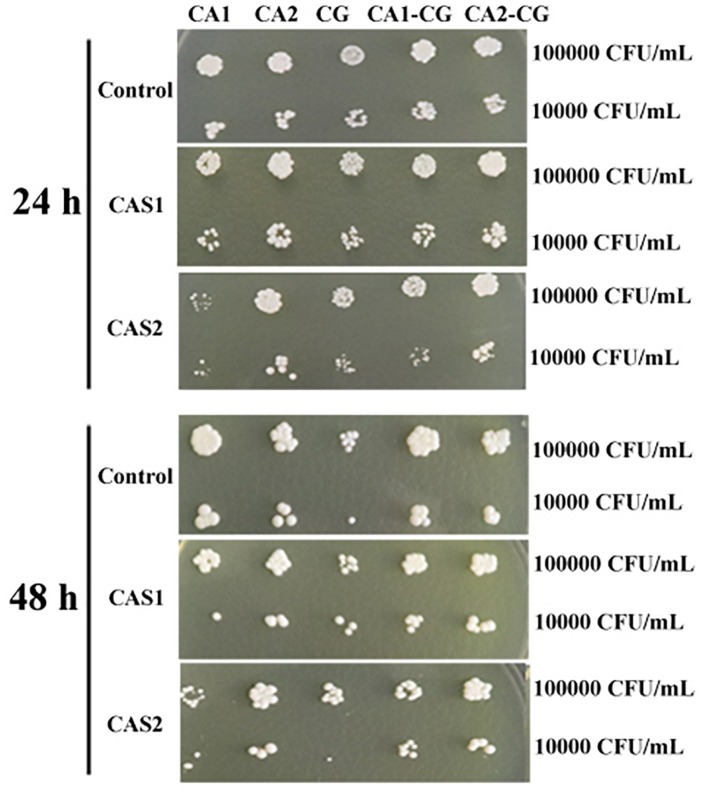
Spot assay of the 24- and 48-h *C. albicans* SC5314 (CA1), *C. albicans* Z2003 (CA2), *C. glabrata* ATCC15126 (CG), *C. albicans* SC5314 – *C. glabrata* ATCC15126 (CA1-CG), *C. albicans* Z2003 – *C. glabrata* ATCC15126 (CA2-CG) mono- and dual-species biofilms on YPD agar containing no drug (Control), 0.2 μg/mL caspofungin (CAS1), and 0.3 μg/mL caspofungin (CAS2) for 24 h at 37°C. The inoculum was 1 × 10^4^ and 1 × 10^5^ CFU/mL.

**FIGURE 4:**
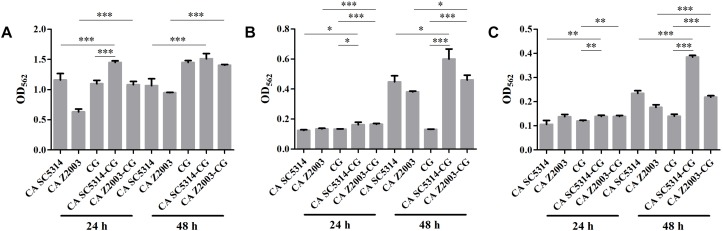
Biofilm biomass quantification of *C. albicans* SC5314 (CA SC5314), *C. albicans* Z2003 (CA Z2003), *C. glabrata* ATCC15126 (CG), *C. albicans* SC5314 – *C. glabrata* ATCC15126 (CA SC5314-CG), *C. albicans* Z2003 – *C. glabrata* ATCC15126 (CA Z2003-CG) single and mixed biofilms treated by **(A)** drug-free control, **(B)** 0.2 μg/mL caspofungin, and **(C)** 0.3 μg/mL caspofungin for 24 and 48 h at 37°C. ^*^p < 0.05; ^∗∗^*p* < 0.01; ^∗∗∗^*p* < 0.001. SB, single biofilm; MB, mixed biofilm.

The XTT assay showed that the SMIC_80_ of caspofungin against *C. albicans* SC5314, *C. albicans* Z2003 and *C. glabrata* ATCC15126 single biofilms were respectively 0.25, 0.55, and 0.30 μg/mL at 24 h and rose up to 0.60, 0.75, and 0.80 μg/mL at 48 h. Accordingly, we chose 0.2 and 0.3 μg/mL caspofungin for spot assay and biomass quantification as they were close to 24-h SMIC_80_. In the spot assay, the inhibitions of 0.3 μg/mL caspofungin on the 24-h *Candida* single biofilms and their mixed counterparts was better than the 0.2 μg/mL caspofungin, the similar results were also observed in the 48-h biofilms (Figure 3). The biofilm biomass was quantified by CV staining under the same conditions in the spot assay. It could be found that in the drug-free control, the biomass of *C. albicans* SC5314/Z2003 – *C. glabrata* ATCC15126 mixed biofilms were significantly higher than their individual counterparts except CG at both 24 and 48 h (*p* < 0.001, Figure 4A). When exposed to 0.2 and 0.3 μg/mL caspofungin, the biomass of the 24- and 48-h *Candida* single and mixed biofilms were suppressed compared with their corresponding counterparts without drug treatment (Figures 4A–C). Further, we also noted that the biomass of caspofungin-treated *C. albicans* SC5314, *C. albicans* Z2003, and *C. glabrata* ATCC15126 single biofilms were remarkably decreased compared with their mixed counterparts at 24 and 48 h (Figures 4B,C), except one case that the biomass of *C. albicans* Z2003 single biofilms was comparable to its mixed counterpart treated by 0.3 μg/mL caspofungin for 24 h (Figure 4C).

### Invasive Proteinase Levels in Single/Mixed *C. albicans* and *C. glabrata* Biofilms

**FIGURE 5:**
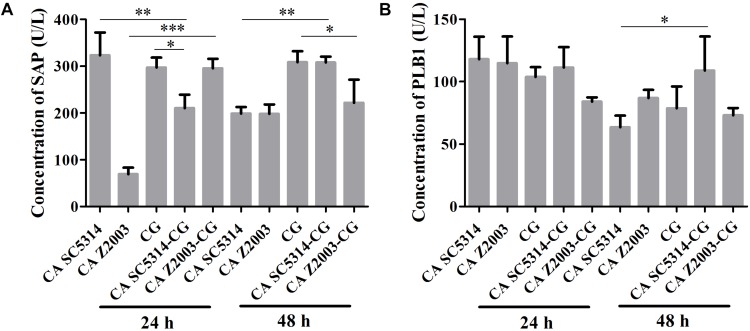
Concentrations of **(A)** secreted aspartic proteinase (SAP) and **(B)** phospholipase B1 (PLB1) in the 24- and 48-h *C. albicans* SC5314 (CA SC5314), *C. albicans* Z2003 (CA Z2003), *C. glabrata* ATCC15126 (CG), *C. albicans* SC5314 – *C. glabrata* ATCC15126 (CA SC5314-CG), *C. albicans* Z2003 – *C. glabrata* ATCC15126 (CA Z2003-CG) single and mixed biofilms by ELISA. ^*^p < 0.05; ^∗∗^*p* < 0.01; ^∗∗∗^*p* < 0.001.

As shown, after 24 h of incubation, the SAP levels in *C. albicans* SC5314 and *C. glabrata* ATCC15126 single biofilms were decreased compared with their mixed counterparts (*p* < 0.01, *p* < 0.05, Figure 5A). In contrast, the SAP in *C. albicans* Z2003 single biofilm was promoted compared with its combination with *C. glabrata* ATCC15126 (*p* < 0.001, Figure 5A). When the incubation time was elongated to 48 h, the SAP level in *C. albicans* SC5314 single biofilm was increased compared with its mixed counterpart with *C. glabrata* ATCC15126 (*p* < 0.01, Figure 5A), while the SAP production in *C. glabrata* ATCC15126 single biofilm was suppressed evidently in comparison with its mixed counterpart with *C. albicans* Z2003 (*p* < 0.05, Figure 5A). As for PLB1, no notable changes were observed in the 24-h *C. albicans* SC5314/Z2003 and *C. glabrata* ATCC15126 single/mixed biofilms. Whereas in the 48-h *C. albicans* SC5314 single biofilm, the PLB1 level was enhanced compared with its mixed counterpart with *C. glabrata* ATCC15126 (*p* < 0.05, Figure 5B).

### R6G Efflux in Single/Mixed *C. albicans* and *C. glabrata* Biofilms

**FIGURE 6:**
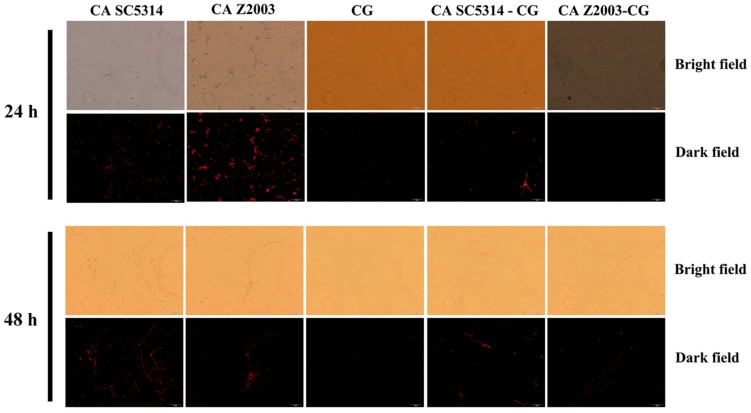
Fluorescent images of rhodamine 6G efflux in the 24- and 48-h *C. albicans* SC5314 (CA SC5314), *C. albicans* Z2003 (CA Z2003), *C. glabrata* ATCC15126 (CG), *C. albicans* SC5314 – *C. glabrata* ATCC15126 (CA SC5314-CG), *C. albicans* Z2003 – *C. glabrata* ATCC15126 (CA Z2003-CG) mono- and dual-species biofilms. Scale bar: 50 μm.

**FIGURE 7:**
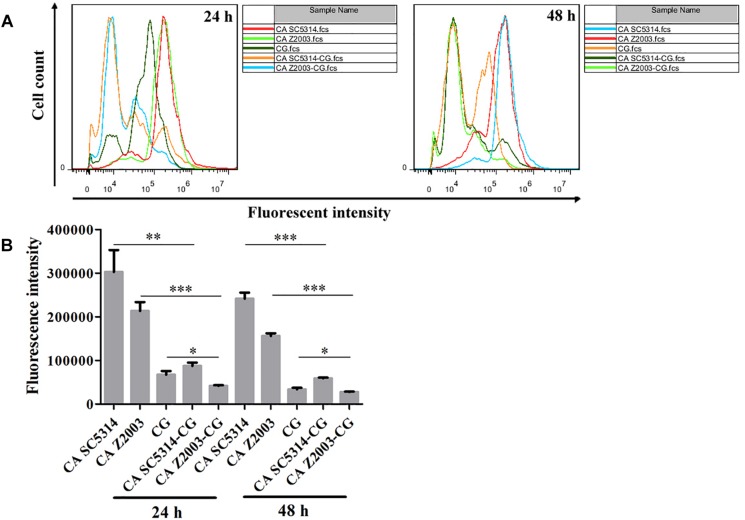
**(A)** Flow cytometry analysis and **(B)** quantification of rhodamine 6G efflux in the 24- and 48-h *C. albicans* SC5314 (CA SC5314), *C. albicans* Z2003 (CA Z2003), *C. glabrata* ATCC15126 (CG), *C. albicans* SC5314 – *C. glabrata* ATCC15126 (CA SC5314-CG), *C. albicans* Z2003 – *C. glabrata* ATCC15126 (CA Z2003-CG) mono- and dual-species biofilms. ^*^p < 0.05; ^∗∗^*p* < 0.01; ^∗∗∗^*p* < 0.001.

The fluorescence of R6G (red) became weak in the 24- and 48-h *C. albicans* SC5314/Z2003 – *C. glabrata* ATCC15126 mixed biofilms compared with their single counterparts (Figure 6). Consistently, the fluorescent intensities in both the 24- and 48-h *C. albicans* SC5314, *C. albicans* Z2003 and *C. glabrata* ATCC15126 single biofilms were markedly reduced compared with their corresponding single counterparts (*p* < 0.05, *p* < 0.01, *p* < 0.001, Figures 7A,B).

### Efflux-Associated Gene Expression in *C. albicans* and/or *C. glabrata* Biofilms

**FIGURE 8:**
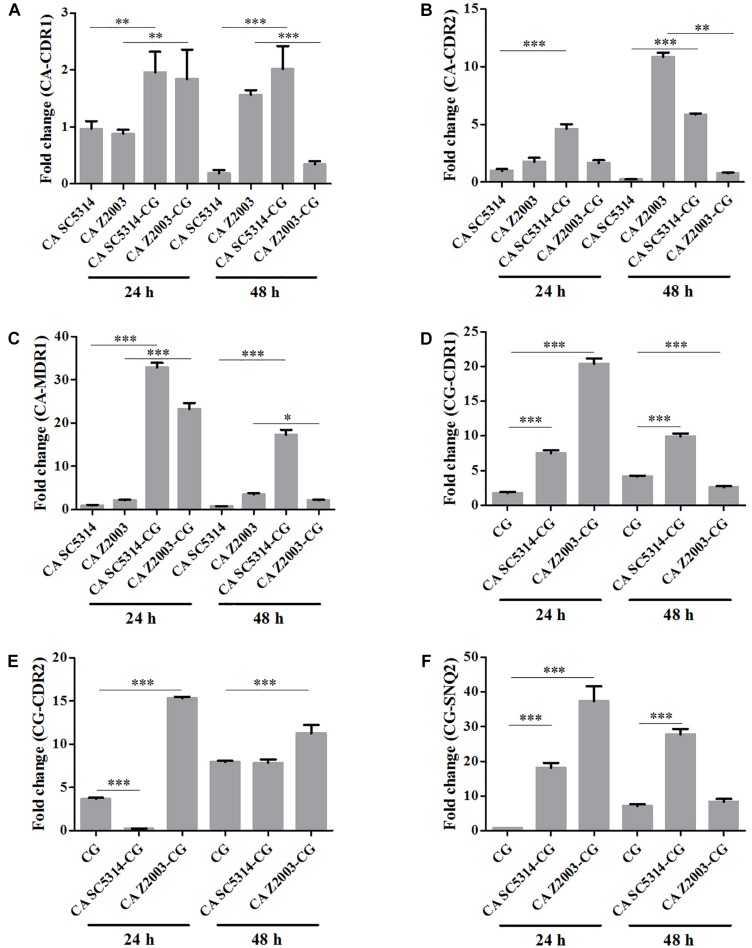
Relative gene expressions of **(A)** CA-CDR1, **(B)** CA-CDR2, **(C)** CA-MDR1, **(D)** CG-CDR1, **(E)** CG-CDR2, **(F)** CG-SNQ2 in the 24- and 48-h *C. albicans* SC5314 (CA SC5314), *C. albicans* Z2003 (CA Z2003), *C. glabrata* ATCC15126 (CG), *C. albicans* SC5314 – *C. glabrata* ATCC15126 (CA SC5314-CG), *C. albicans* Z2003 – *C. glabrata* ATCC15126 (CA Z2003-CG) mono- and dual-species biofilms. The *CDR1* mRNA of the 24-h *C. albicans* SC5314 single biofilm was set as the control. ^*^p < 0.05; ^∗∗^*p* < 0.01; ^∗∗∗^*p* < 0.001.

The relative mRNA expressions of the major efflux-associated genes were analyzed in the 24- and 48-h *Candida* single and mixed biofilms with the *CDR1* mRNA of the 24-h *C. albicans* SC5314 single biofilm as the control. In the 24-h *Candida* single/mixed biofilms, the *CDR1*, *CDR2* and *MDR1* levels in *C. albicans* SC5314/Z2003 single biofilm, and the *CDR1*, *CDR2* and *SNQ2* levels in *C. glabrata* ATCC15126 single biofilm were increased strikingly compared with their mixed counterparts (*p* < 0.001 and *p* < 0.01, Figures 8A–F) except the *CDR2* expression in *C. albicans* Z2003 and *C. glabrata* ATCC15126 single biofilms when respectively being compared with *C. albicans* Z2003 – *C. glabrata* ATCC15126 and *C. albicans* SC5314 – *C. glabrata* ATCC15126 mixed counterparts (Figures 8B,E). For the 48-h *Candida* single/mixed biofilms, the *CDR1*, *CDR2*, and *MDR1* expressions in *C. albicans* SC5314 single biofilm still displayed significant increases compared with its mixed counterparts (*p* < 0.001, Figures 8A–C). On the contrary, the three gene expressions in *C. albicans* Z2003 single biofilm were suppressed largely compared with its mixed counterparts (Figures 8A–C). In *C. glabrata* ATCC15126 single biofilm, the conspicuous upregulations of the CG-*CDR1* and CG-*SNQ2* could be observed compared with *C. albicans* SC5314 – *C. glabrata* ATCC15126 mixed biofilms (*p* < 0.001, Figures 8D,F) and the CG-*CDR2* level was unchanged (Figure 8E). Whereas the CG-*CDR1* experienced downregulation (*p* < 0.001, Figure 8D), upregulation (*p* < 0.001, Figure 8E), and no change (Figure 8F) compared with *C. albicans* Z2003 – *C. glabrata* ATCC15126 mixed biofilms.

### β-Glucan Exposure in Single/Mixed *C. albicans* and *C. glabrata* Biofilms

**FIGURE 9:**
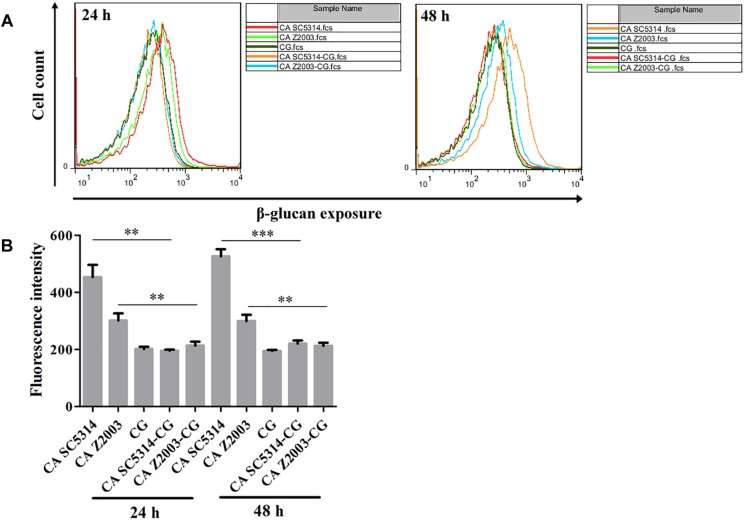
**(A)** Flow cytometry analysis and **(B)** quantification of β-glucan exposure in the 24- and 48-h *C. albicans* SC5314 (CA SC5314), *C. albicans* Z2003 (CA Z2003), *C. glabrata* ATCC15126 (CG), *C. albicans* SC5314 – *C. glabrata* ATCC15126 (CA SC5314-CG), *C. albicans* Z2003 – *C. glabrata* ATCC15126 (CA Z2003-CG) mono- and dual-species biofilms. ^*^p < 0.05; ^∗∗^*p* < 0.01; ^∗∗∗^*p* < 0.001.

As exhibited, the β-glucan exposure was remarkably declined in *C. albicans* SC5314/Z2003 single biofilm compared with their corresponding mixed counterparts with *C. glabrata* ATCC15126 (*p* < 0.01, *p* < 0.001, Figures 9A,B). Whereas the exposure extent of β-glucan was comparable between the 24- and 48-h *C. glabrata* ATCC15126 single biofilms and their corresponding mixed counterparts (Figures 9A,B).

## Discussion

In the surroundings, interspecific interactions between two or more microorganisms are prevailing, and so is the same case in human body. In clinical context, the most suspicious pathogen is identified and blamed for the cause of a type of infection even though the body is encompassed by a complex microbiome. In terms of this fact, the incidence of mixed microbial infections is possibly, to a large extent, understated (Peters et al., 2012).

*Candida albicans* and *C. glabrata* are two commonly opportunistic pathogens and can be frequently found together in oral cavity, but the isolation of the latter alone is scarce from the infection sites (Silva et al., 2011), which might be likely due to the lower virulence in mono-invasion of *C. glabrata* (Tati et al., 2016). In our previous effort, the robust *C. albicans* and *C. glabrata* mixed biofilms were formed under static and flow states in a home-made device for dynamic biofilm preparation. Our study and most other reports showed that *C. albicans* developed a multilayer and dense biofilm with intricate hyphae as a scaffold for *C. glabrata* attachment (Silva et al., 2013; Shao et al., 2015; Santos et al., 2016; Hosida et al., 2018; Olson et al., 2018). This synergistic interaction between *C. albicans* and *C. glabrata* is, to a large extent, different from that between *C. albicans* and *C. krusei*, in which the characteristic “long grain rice”-shaped blastospore of *C. krusei* is opposed to the hyphal elements of *C. albicans* (Thein et al., 2007). However, the synergism between *C. albicans* and *C. glabrata* might be associated with their competition upon growth found in limited *Candida* strains (Silva et al., 2013; Rossoni et al., 2015). Following expansion to 16 couples of *C. albicans* and *C. glabrata* strains, consistently, we observed that the growth of the two *Candida* species in their both 24- and 48-h mixed biofilms seemed to slow down with marked decrease of individual cell counting compared with their mixed counterparts (Figures 1, 2). These results were in agreement with the previous results and confirmed the competitive interaction between *C. albicans* and *C. glabrata* when they were incubated concurrently at least within 48 h. The 24- and 48-h cell proportions of *C. albicans* and *C. glabrata* could provide a dynamic variation of relative abundance manifesting the advantageous strain compared with their corresponding single biofilms. At 24 h, 4 in 8 groups in both *C. albicans*-*C. glabrata* ATCC15126 and *C. albicans*-*C. glabrata* ATCC28226 mixed biofilms showed increased cell proportion indicating that the four *C. albicans* strains in the corresponding mixed biofilms were more competitive than *C. glabrata* (Tables 1, 2). Whereas at 48 h, 8 in 8 groups in *C. albicans*-*C. glabrata* ATCC15126 and 7 in 8 groups in *C. albicans* – *C. glabrata* ATCC28226 mixed biofilms displayed increased cell proportion indicating that nearly all of the test *C. albicans* strains in the corresponding mixed biofilms presented a more strong competitiveness than *C. glabrata* (Tables 1, 2).

Most of the published data support that *C. albicans* and *C. glabrata* mixed biofilms (either co-cultured simultaneously or co-incubated of *C. glabrata* with preformed *C. albicans* biofilm) can favorably colonize and be more aggressive against host tissues indicative of increased virulence of mixed biofilms (Tati et al., 2016; Olson et al., 2018). Although there was a demonstration that the reduced virulence of *C. albicans* and *C. glabrata* mixed biofilms was proportional to the decline of individual cell number of *C. albicans* and *C. glabrata* (Rossoni et al., 2015), a more confirmative conclusion requires the expansion of *Candida* strains especially from clinical sources and deep investigation on the comparison of virulence between mono-*Candida* biofilms and their mixed counterparts. Thus, a group of virulence factors were evaluated in the *C. albicans* SC5314/*C. albicans* Z2003 and *C. glabrata* ATCC 15126 single/mixed biofilms in this study.

Caspofungin is currently the first-line agents with efficiently antifungal activity and favorably low cytotoxicity (Larkin et al., 2018). Both spot assay and CV staining demonstrated that the susceptibilities of *Candida* mixed biofilms to caspofungin were not impaired compared with their single counterparts (Figures 3, 4), indicating that the competition between *C. albicans* and *C. glabrata* leading to the decline of population quantity seemed to be not at the expense of drug resistance.

There is a family of at least 10 genes that are responsible to encode SAP (*SAP1*-*SAP10*) and four genes involved in PL production (*PLA*-*PLD*) in which *PLB1* appears to be a major contributor for PL activity in *C. albicans* associated with adhesion, invasion, and tissue damage (Ghannoum, 2000; Silva et al., 2014). In this study, the SAP and PLB1 levels were varying and presented heterogeneity that might be dependent on strains and experimental conditions. In contrast to *C. albicans*, no SAP gene has been identified and no study related with PL was reported in *C. glabrata* (Marcos-Arias et al., 2011; Silva et al., 2012, 2014) in spite of a relevant report that uncovered the production of unspecified proteinase (Chakrabarti et al., 1991). It is unclear why in *C. glabrata* ATCC15126, there had measurable SAP and PLB1 levels in opposite to the documents aforementioned. We suppose that the glycosylphosphotidylinositol (GPI) anchored aspartic proteinases and unknown extracellular proteinases might contribute to the unexpected results (Chakrabarti et al., 1991; Kaur et al., 2007). As the invasion of *Candida* strains rely on secretion of many types of extracellular proteinases including SAP and PLB1, it is reasonable to consider these proteinases as a whole when inspecting the impact of decreased cell number on *Candida* virulence in single and mixed biofilms.

Rhodamine 6G is a widely-used fluorescent dye for monitoring the function of efflux pumps, and the experimental process requires the addition of glucose as energy (Peralta et al., 2012). In this study, the fluorescent results exhibited that R6G was efficiently pumped out of the fungal cells compared the 24-/48-h single *Candida* biofilms with their mixed counterparts. These results were in good agreement with the fluorescent images, suggestive of the activated transporters along with the decreased fungal cell quantity.

Two major efflux pumps, ABC superfamilies and MFS pumps, are known to mediate the resistance of *Candida* species, in which ABC superfamilies is driven by ATP hydrolysis and MFS pumps needs the proton-motive force across the membrane (Cannon et al., 2009). The most widely studied genes associated with efflux function are *CDR1*, *CDR2*, and *MDR1* in *C. albicans*, while in *C. glabrata*, they generally consist of *CDR*1, *CDR*2 (*PDH*1), and *SNQ*2 (Sanglard et al., 1999, 2001; Torelli et al., 2008). The six efflux-associated genes (CA-*CDR1*, CA-*CDR2*, CA-*MDR1*, CG-*CDR*1, CG-*CDR*2, and CG-*SNQ*2) were upregulated significantly when comparing the 24-/48-h *C. albicans* SC5314 and *C. glabrata* ATCC15126 single biofilms with their mixed counterpart, revealing that the efflux pumps were more active in the mixed biofilms than the single ones consistent with the rhodamine 6G efflux results. The similar conclusion seemed to be proper in the 24-h *C. albicans* Z2003/*C. glabrata* ATCC15126 single and mixed biofilms. In contrast, the six genes except CG-*CDR*2 were all downregulated comparing the 48-h *C. albicans* Z2003 and *C. glabrata* ATCC15126 single biofilms with their mixed counterpart, assuming that CG-*CDR2* might become a major contributor to mediating the efflux pump function between 24 and 48 h and other transporter-related genes that have not been measured in this study might be involved in the normal function of *C. albicans* and *C. glabrata* efflux pumps (Maesaki et al., 1999; Costa et al., 2016). These results further demonstrated that the going-down of cell number did not affect the efflux pump normal function in the single and mixed *C. albicans* and *C. glabrata* biofilms.

β-Glucan, a critical component in the *Candida* cell wall, is usually buried underneath mannoprotein exoskeleton and cannot be recognized by the innate immune cells. Once the *Candida* species are inflicted with external stresses (such as drug attack), β-glucan appears in the outer cell wall surface and becomes a potent stimulator of the immune response to facilitate antifungal recognition, the dynamic process of which is called β-glucan unmasking, a part of cell wall remodeling/rearrangement (Hopke et al., 2018). Reasonably, the exposure extent of β-glucan, a crucial cell wall component, is correlated with *Candida* invasion potential (Ballou et al., 2016). Our results showed that the unmasking of β-glucan was suppressed in the 24- and 48-h *C. albicans* – *C. glabrata* mixed biofilms compared with their 24- and 48-h *C. albicans* counterparts, suggesting enhanced immune recognition of the *Candida* mixed biofilms as opposed to the *C. albicans* single biofilms. Although it is hard to distinguish the individual β-glucan exposure extent of *C. albicans* SC5314/2003 and *C. glabrata* ATCC15126 in their mixed biofilms, the reduced β-glucan unveiling indicated that *C. glabrata* could help *C. albicans* remodel its cell wall to mask unnecessary β-glucan if considering the comparable fluorescent intensities between the 24-/48-h *C. glabrata* ATCC15126 mono-biofilm and *C. albicans* SC5314/Z2003 – *C. glabrata* ATCC15126 dual-biofilms.

## Conclusion

We demonstrate that *C. albicans* and *C. glabrata* can grow together and form mixed biofilm with competition, the virulence of the mixed biofilms is not weakened compared with their single counterparts in terms of the dramatic shrinkage of each *Candida* species population (at least within 48 h of incubation). However, whether the factors including competitive adhesion/colonization inhibition, diffusive molecular mediators as well as nutritional deprivation contribute to the underlying mechanism in the formation of *C. albicans* and *C. glabrata* dual-species biofilm needs more evidence. Deciphering the interaction of *C. albicans* and *C. glabrata* will not only expand our understanding of the development law of other *Candida* mixed biofilms, but also shed light on the pathogenesis of mixed *Candida* invasion and the host immune response to interspecific biofilms.

## Data Availability

The raw data supporting the conclusions of this manuscript will be made available by the authors, without undue reservation, to any qualified researcher.

## Author Contributions

QL, JL, and WD performed the experiment. JS, GS, and TW analyzed the data. JS and DW wrote the manuscript. JS and CW devised the experiment.

## Conflict of Interest Statement

The authors declare that the research was conducted in the absence of any commercial or financial relationships that could be construed as a potential conflict of interest.
